# Right Atrial Mass in a Patient With HIV and Hepatitis B: A Case Report

**DOI:** 10.4021/cr293w

**Published:** 2013-10-15

**Authors:** Muhammad Umer Siddiqui, Masroor khan, Timithy Anderson

**Affiliations:** a Internal Medicine, Mount Sinai Hospital-Englewood program, 151 engle st Apt A11 englewood NJ 07631, USA; b Interventional Cardiology, 10021 S Main St suite B-1 Houston TX 77025, USA; c Park Plaza Hospital Houston TX 77004, USA

**Keywords:** Atrial Mass, Hepato-cellular carcinoma metastasis, Hepatocellular carcinoma and atrial mass

## Abstract

A 41-year-old man presented to the emergency room for evaluation of substernal chest pain, shortness of breath and generalized failure to thrive. Patient had history of hepatitis B and HIV. During recent evaluation of hepatic mass, patient was found to have hepatocellular carcinoma on biopsy. Patient had no history of cirrhosis of the liver in the past. On Echocardiogram patient was noted to have a large mass filling the right atrial cavity. CT scan of abdomen, pelvis and chest showed a diffusely enlarged heterogeneously enhancing liver consistent with large hepatoma, with portal venous and hepatic vein thrombosis. Tumor thrombus extended through the hepatic veins and upper inferior vena cava into the right atrium. There was 6 cm greatest diameter enhancing mass in the right atrium. Patient had primary hepatocellular carcinoma with extensive invasion into vascular structures. His prognosis was poor and patient opted for palliative care only. In conclusion, patients with co-infection of HIV and Hepatitis B are at risk of developing hepatocellular carcinoma with extension into the right atrium and physicians managing these patients should have high suspicion of right atrial involvement with tumor extension and low threshold to order a screening echocardiogram.

## Introduction

Metastatic involvement of the heart is much more common than primary cardiac tumors. One such tumor metastasizing to the heart is hepatocellular carcinoma. Cardiac tumors may be asymptomatic or found incidentally during evaluation. Signs and symptoms are usually determined by the location of the tumor in the heart. Common symptoms in a patient with cardiac tumors are difficulty breathing, lightheadedness, palpitations and chest pain. A mass in the heart can be detected by echocardiography, MRI or a CT scan. Hepatocellular carcinoma has a tendency to metastasize to the heart. Studies have indicated that patients co-infected with HIV and Hepatitis B have more chances of developing hepatocellular carcinoma and ultimately leading to heart metastasis than a mono-infected patient. We have described here a case of hepatocellular carcinoma leading to heart metastasis.

## Case Report

A 41-year-old man presented to the emergency room for evaluation of substernal chest pain, shortness of breath and generalized failure to thrive. Patient had history of hepatitis B and HIV being treated with Truvada and Kaletra. During recent evaluation on outpatient basis for hepatic mass, patient was found to have hepatocellular carcinoma on biopsy. Patient had no history of cirrhosis of the liver in the past. Heart examination was benign. Abdomen was distended with tenderness to deep palpation on right upper quadrant.

**Figure 1:**
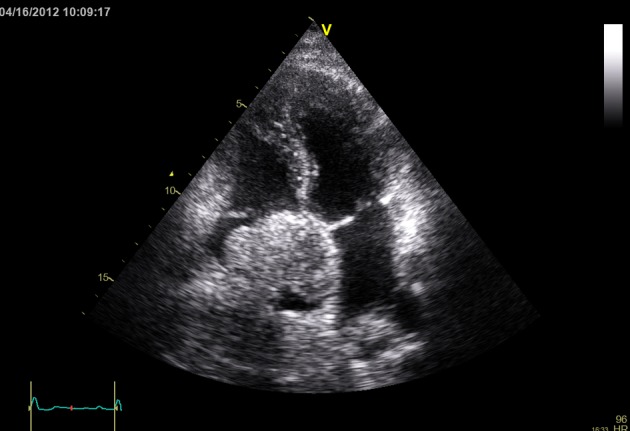
Four chamber view with right atrial mass.

**Figure 2:**
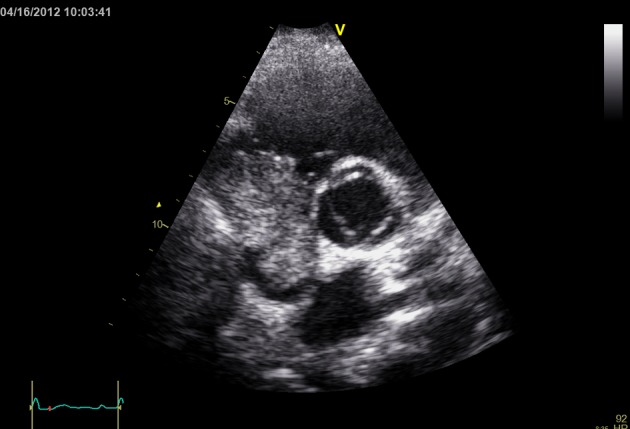
Short axis view at aortic valve showing right atrial mass.

On Echocardiogram patient was noted to have a large mass filling the right atrial cavity (Fig. 1, 2). CT scan of abdomen, pelvis and chest showed a diffusely enlarged heterogeneously enhancing liver consistent with large hepatoma, with portal venous and hepatic vein thrombosis. Tumor thrombus extended through the hepatic veins and upper inferior vena cava into the right atrium. There was 6 cm greatest diameter enhancing mass in the right atrium. CBC showed increased neutrophils and decreased lymphocytes. Patient had non-detectable viral loads with normal liver enzymes except Aspartate Aminotransferase (AST) which was mildly elevated. Liver Function Tests further showed decreased albumin levels and mildly increased total bilirubin.

Patient had primary hepatocellular carcinoma with extensive invasion into vascular structures. His prognosis was poor and patient opted for palliative care only.

## Discussion

Metastasis to the heart is much more common than primary cardiac tumors and is generally associated with a poor prognosis. Tumors that are most likely to involve the heart and pericardium include cancers of the lung and breast, melanoma, lymphoma and liver [1]. Hepatocellular carcinoma has a great tendency towards venous invasion; however, extension of metastatic hepatocellular carcinoma into the right atrium was rarely reported [2-5]. Prognosis of hepatocellular carcinoma with cardiac metastasis is poor with median survival ranging from 1 - 4 months [6].

Our case is unique in a way that this reports HIV/Hepatitis B associated hepatocellular carcinoma metastasizing to the right atrium. In the US approximately 10% of patients with HIV are co-infected with chronic hepatitis B. Once chronic Hepatitis B is established, HIV affects hepatitis B e antigen (HBeAg) clearance, HBV replication and liver disease [7-9].

There are limited data on whether HIV affects the development of hepatocellular carcinoma. One French study of 822 HIV infected individuals of whom 8% were co infected with hepatitis B found that 22% of deaths in the HIV-HBV co infected group were liver related compared to 2% in the HIV mono infected group [10]. In the HIV-HBV co infected group, 50% of the liver related deaths were from hepatocellular carcinoma compared to 13% of the liver related deaths in the HIV mono infected group. This study suggests that HIV may accelerate the development of HBV related hepatocellular carcinoma. The Swiss HIV cohort study found that for every 100 cell/mL decrease in CD-4 T cell count there was a 1.33 fold increase risk of developing hepatocellular carcinoma (95% CI 1.06 - 1.68) [11].

Anti-retroviral therapy (ART) may also affect the natural history of hepatitis B. Several studies have demonstrated that HIV-HBV co-infected patients have an increased incidence of hepato-toxicity (defined as aminotransferase elevation) from ART compared to HIV mono infected patients [12, 13].

The studies clearly show that HIV has a negative impact on hepatitis B infection including decreased immune response and accelerated liver disease. Furthermore, ART also leads to increase hepato-toxicity in chronic hepatitis B. Therefore, patients with HIV-HBV co-infection should be screened with serial radiologic imaging of liver along with AFP, to look for the development of hepatocellular carcinoma and therapy with least toxic affect on liver should be started.

The prevalence of right atrium involvement in hepatocellular carcinoma patients was reported to be 2.4-6.3% [14, 15]. A screening examination using trans-esophageal echocardiography in hepatocellular carcinoma patients even showed the prevalence of subclinical cardiac metastasis in hepatocellular carcinoma patients may be as high as 11% [16] and therefore we suggest that patients presenting with hepatocellular carcinoma and shortness of breath should get 2D Echocardiography.
